# Real-World Comparative Survival Outcomes of First-Line Serplulimab Plus Chemotherapy in Extensive-Stage Small-Cell Lung Cancer: A Single-Center Retrospective Cohort Study

**DOI:** 10.7150/jca.140183

**Published:** 2026-10-16

**Authors:** Yuhao Jing, Miao Li, Songxiang Li, Yifan Zhao, Zhenbao Zhou, Xin Li, Meng Wang

**Affiliations:** 1 School of Medicine, Tianjin University, Tianjin, China.; 2 Department of Thoracic Surgery, Tianjin University Haihe Hospital, Tianjin 300350, China.; 3 Department of Thoracic Surgery, Tianjin Chest Hospital (Affiliated Hospital of Tianjin University), Tianjin 300222, China.

**Keywords:** extensive-stage small-cell lung cancer, serplulimab, chemoimmunotherapy, real-world evidence, overall survival, overlap weighting

## Abstract

**Background:**

Extensive-stage small-cell lung cancer (ES-SCLC) remains an aggressive malignancy with poor survival despite platinum-etoposide chemotherapy. Although serplulimab plus chemotherapy has demonstrated a survival benefit in randomized trials, comparative real-world evidence remains limited. We therefore evaluated survival outcomes with first-line serplulimab plus chemotherapy versus chemotherapy alone in patients with ES-SCLC.

**Methods:**

We conducted a single-center retrospective cohort study at Tianjin Chest Hospital. Patients with ES-SCLC who initiated first-line platinum-etoposide-based chemotherapy between January 2020 and December 2024 were screened, with follow-up censored on May 1, 2025. Patients were classified according to whether serplulimab was incorporated into first-line treatment. The primary endpoint was overall survival (OS), and the secondary endpoint was real-world progression-free survival (rwPFS). Treatment effects were estimated using Kaplan-Meier analyses and Cox proportional hazards models. Because of the limited sample size and event count, the primary adjusted Cox model was restricted to prespecified clinical covariates. Sensitivity analyses included treatment-year adjustment, restriction to patients treated during 2022-2024, reduced propensity score overlap weighting and stabilized trimmed inverse probability of treatment weighting (IPTW).

**Results:**

The final cohort included 112 patients, of whom 30 received serplulimab plus chemotherapy and 82 received chemotherapy alone. Median OS was 18.69 months with serplulimab plus chemotherapy and 11.86 months with chemotherapy alone (HR = 0.49, 95% CI: 0.27-0.90; P = 0.021; log-rank P = 0.017). The association remained significant after parsimonious clinical adjustment (adjusted HR = 0.50, 95% CI: 0.27-0.93; P = 0.028). Sensitivity analyses yielded directionally similar OS estimates across treatment-year adjustment, 2022-2024 restriction, overlap weighting, and stabilized trimmed IPTW. Median rwPFS was also numerically longer with serplulimab plus chemotherapy, although the difference was not statistically significant (10.64 vs 8.18 months; HR = 0.76, 95% CI: 0.48-1.20; P = 0.244).

**Conclusions:**

In this single-center real-world cohort, first-line serplulimab plus chemotherapy was associated with longer OS than chemotherapy alone in patients with ES-SCLC, whereas the rwPFS difference was not statistically significant. The findings were directionally supported by multiple sensitivity analyses but should be interpreted cautiously because of the limited sample size, potential calendar-time confounding, and incomplete standardized safety assessment. Larger prospective studies are needed for confirmation.

## Introduction

Small-cell lung cancer (SCLC) is an aggressive neuroendocrine malignancy characterized by rapid tumor growth, early metastatic dissemination, and poor prognosis 1-4. Most patients present with extensive-stage disease, for which curative treatment is rarely feasible 3,5. Although extensive-stage SCLC (ES-SCLC) is initially highly sensitive to chemotherapy, relapse is common and long-term survival remains poor 5,6.

For decades, platinum-etoposide chemotherapy was the standard first-line treatment for ES-SCLC 5,6. Although this regimen often induces rapid tumor responses, disease control is typically short-lived and survival outcomes remain limited 5,6. The incorporation of immune checkpoint inhibitors into first-line therapy has subsequently changed the treatment landscape. Randomized clinical trials have demonstrated that adding immune checkpoint blockade, including atezolizumab or durvalumab, to platinum-etoposide chemotherapy improves survival compared with chemotherapy alone 7-11. These findings established chemoimmunotherapy as a standard first-line treatment strategy for ES-SCLC 11-19.

Serplulimab is a programmed cell death protein 1 (PD-1) inhibitor that has demonstrated survival benefit in treatment-naïve ES-SCLC 13-15. In the phase 3 ASTRUM-005 trial, serplulimab plus platinum-etoposide chemotherapy significantly improved overall survival compared with chemotherapy alone 13-15. These results support serplulimab-based chemoimmunotherapy as an important first-line option for ES-SCLC 13-15,19. However, comparative real-world evidence for serplulimab remains limited, particularly regarding whether the survival benefit observed in randomized trials is reproduced in routine clinical practice 20-26.

Real-world evaluation is particularly relevant because patients treated outside clinical trials may differ in age, comorbidity burden, metastatic patterns, supportive care, and follow-up intensity 20-26. In addition, retrospective treatment comparisons are susceptible to baseline imbalance, non-random treatment selection, calendar-time effects, and incomplete outcome ascertainment. Accordingly, analyses incorporating multivariable adjustment and complementary sensitivity approaches are important for evaluating the consistency of observed treatment associations.

In this study, we conducted a single-center retrospective cohort study at Tianjin Chest Hospital to compare survival outcomes between first-line serplulimab plus chemotherapy and chemotherapy alone in patients with ES-SCLC. The primary endpoint was overall survival (OS), and the secondary endpoint was real-world progression-free survival (rwPFS). We further assessed the consistency of the OS association using multivariable adjustment, calendar-period restriction, overlap weighting, inverse probability of treatment weighting (IPTW), and exploratory subgroup analyses.

## Methods

### Study design and patients

This was a single-center retrospective cohort study conducted at Tianjin Chest Hospital, a tertiary referral center specializing in thoracic malignancies. Patients with extensive-stage small-cell lung cancer (ES-SCLC) who initiated first-line platinum-etoposide-based chemotherapy between January 2020 and December 2024 were screened for eligibility. The data cutoff date was May 1, 2025.

### Eligibility criteria

Patients were eligible if they met all of the following criteria: (1) age ≥18 years; (2) histologically or cytologically confirmed small-cell lung cancer (SCLC); (3) extensive-stage disease according to the Veterans Administration Lung Study Group (VALSG) staging system^5^, ^19^; (4) receipt of first-line platinum-etoposide-based chemotherapy with or without serplulimab; and (5) availability of complete baseline clinical information and follow-up data for overall survival (OS) and real-world progression-free survival (rwPFS).

Patients were excluded if they met any of the following criteria: (1) absence of histologic or cytologic confirmation of small-cell lung cancer, uncertain pathologic diagnosis, or a diagnosis suggesting another primary thoracic malignancy; (2) limited-stage small-cell lung cancer at the initiation of first-line treatment, or inability to confirm extensive-stage disease from the available medical records; (3) receipt of prior systemic therapy for small-cell lung cancer before the index first-line regimen, unclear treatment line, or treatment not initiated as first-line therapy; (4) receipt of a non-standard first-line regimen, including regimens not based on platinum-etoposide chemotherapy, use of immune checkpoint inhibitors other than serplulimab in the first-line setting, or inability to reliably classify treatment as serplulimab plus chemotherapy or chemotherapy alone; (5) presence of another active malignancy or severe comorbidity expected to substantially affect survival assessment; or (6) incomplete key medical records, including missing baseline clinical information, treatment initiation date, treatment regimen, progression or death status, event date, or last follow-up date. Patients meeting more than one exclusion criterion were categorized according to the primary reason for exclusion during medical record review.

### Data sources and follow-up

Clinical data were extracted from inpatient and outpatient electronic medical records (EMRs), including admission and discharge summaries, oncology clinic notes, laboratory and imaging reports, chemotherapy prescriptions, infusion records, and follow-up documentation. Baseline demographic, clinical, laboratory, treatment, and survival information was independently reviewed and verified by trained investigators.

Survival status and disease progression were ascertained using EMRs, imaging reports, outpatient follow-up records, and structured telephone follow-up when necessary. For patients without an event before the data cutoff, survival time was censored at the date of the last verifiable clinical contact. The final data cutoff date was May 1, 2025.

### Treatment groups

Patients were classified according to the first-line treatment received. The serplulimab plus chemotherapy group included patients who received serplulimab in combination with platinum-etoposide-based chemotherapy as first-line therapy. The chemotherapy-alone group included patients who received platinum-etoposide-based chemotherapy without serplulimab. The chemotherapy backbone consisted of etoposide plus a platinum agent, mainly carboplatin or cisplatin. Treatment selection was determined by physician discretion in routine clinical practice and was not randomized.

### Outcomes

The primary endpoint was overall survival (OS), defined as the time from initiation of first-line therapy to death from any cause or last follow-up.

The secondary endpoint was real-world progression-free survival (rwPFS), defined as the time from initiation of first-line therapy to documented disease progression, death from any cause, or last follow-up, whichever occurred first. Disease progression was abstracted from radiology reports, outpatient and inpatient medical records, and treating physician documentation. Because tumor assessments were performed in routine clinical practice rather than according to a protocol-mandated imaging schedule, this endpoint was defined as rwPFS rather than protocol-defined progression-free survival (PFS). This real-world endpoint approach is consistent with prior frameworks for real-world tumor progression assessment 27,28. When measurable lesion information was explicitly available in the medical records or radiology reports, the Response Evaluation Criteria in Solid Tumors version 1.1 (RECIST 1.1) criteria were used; however, no blinded independent central radiologic review was performed 29.

Second-line and subsequent systemic therapies after documented disease progression were retrospectively ascertained from inpatient medication-order records and supplemented by available clinical documentation. Post-progression therapy was defined as systemic anticancer treatment initiated after documented disease progression. Maintenance serplulimab administered before progression as part of the first-line treatment strategy was not classified as subsequent-line therapy. Because rwPFS events included either progression or death, subsequent-treatment patterns were summarized among patients with documented progression. Treatment categories were not mutually exclusive.

Because prospectively collected CTCAE-graded adverse events and standardized irAE assessments were unavailable, safety was evaluated using predefined record-based proxies. Explicit adverse-event terms were abstracted from available clinical documentation, and systemic corticosteroid exposure potentially consistent with treatment of an immune-mediated event was identified from medication records using a prespecified algorithm. Treatment interruption, permanent treatment discontinuation, toxicity-related hospitalization, and fatal toxicity could not be reliably ascertained from the available retrospective data and were therefore not coded as absent when undocumented. These analyses were descriptive and were not intended to estimate the incidence of treatment-related adverse events.

### Statistical analysis

Continuous variables were summarized as median and interquartile range, and categorical variables were summarized as number and percentage. Baseline balance between treatment groups was assessed using standardized mean differences (SMDs) rather than hypothesis testing, because P values are highly dependent on sample size and do not quantify the magnitude of covariate imbalance. An SMD greater than 0.1 was considered indicative of meaningful imbalance.

Survival curves for OS and rwPFS were estimated using the Kaplan-Meier method and compared using the log-rank test 30. Cox proportional hazards regression models were used to estimate hazard ratios (HRs) and 95% confidence intervals (CIs) 31. The primary treatment effect was first estimated using an unadjusted Cox model. To limit model overfitting in this modest-sized retrospective cohort, the primary multivariable Cox model was deliberately restricted to a small number of prespecified clinically relevant covariates, including age, ECOG performance status, liver metastasis, and brain metastasis. Models incorporating additional laboratory biomarkers, including LDH, NSE, SII, and PNI, were considered exploratory extended adjustment analyses rather than primary adjusted estimates. The proportional hazards assumption was assessed using Schoenfeld residual-based tests 32.

To evaluate the robustness of the OS findings, sensitivity analyses included treatment-year adjustment, combined parsimonious clinical plus treatment-year adjustment, restriction to patients treated during 2022-2024, and parsimonious clinical adjustment within the restricted cohort. Propensity score-based analyses were conducted as sensitivity analyses rather than as primary causal estimates 33,34. To avoid excessive model complexity and unstable weights, a reduced propensity score model including age, ECOG performance status, liver metastasis, and brain metastasis was used. Overlap weighting was selected as the primary propensity score-based approach because it down-weights patients with extreme treatment probabilities and focuses on patients with clinical equipoise 35-37. Stabilized trimmed inverse probability of treatment weighting (IPTW) was performed as an additional sensitivity analysis 34,38. Covariate balance before and after weighting was assessed using SMDs.

Median follow-up time was estimated using the reverse Kaplan-Meier method. Exploratory subgroup analyses for OS were conducted across selected clinical subgroups, including age, sex, ECOG performance status, brain metastasis, liver metastasis, and bone metastasis. Treatment-by-subgroup interaction was assessed using likelihood-ratio tests comparing Cox models with and without interaction terms, and one P-interaction value was reported for each covariate. Laboratory-marker subgroup analyses based on cohort-specific median cutoffs were reported only as supplementary exploratory analyses. This study was reported in accordance with the Strengthening the Reporting of Observational Studies in Epidemiology (STROBE) statement 39.

## Results

### Patient selection and baseline characteristics

A total of 282 patients with extensive-stage small-cell lung cancer who received at least one cycle of platinum-etoposide-based chemotherapy at Tianjin Chest Hospital between January 2020 and December 2024 were screened. After medical-record review, 170 patients were excluded: 11 because of absent or uncertain pathological confirmation; 24 because of limited-stage disease or unconfirmed extensive-stage disease; 26 because of prior systemic therapy or an unclear treatment line; 27 because of a non-standard first-line regimen or inability to classify the first-line treatment group; 39 because of another active malignancy or severe comorbidity expected to substantially affect survival assessment; and 43 because of incomplete key medical records. The final analytic cohort therefore comprised 112 patients, including 30 who received first-line serplulimab plus chemotherapy and 82 who received chemotherapy alone (Figure 1).

During follow-up, 73 deaths and 97 rwPFS events were observed. The chemotherapy-alone group had 60 deaths and 72 rwPFS events, whereas the serplulimab plus chemotherapy group had 13 deaths and 25 rwPFS events. Median follow-up, estimated using the reverse Kaplan-Meier method, was 18.76 months overall, 23.18 months in the chemotherapy-alone group, and 16.29 months in the serplulimab plus chemotherapy group.

Baseline characteristics are summarized in Table 1. The median age of the overall cohort was 68 years, and 92 patients (82.1%) were male. ECOG performance status was 0 in 63 patients (56.2%) and 1 in 49 patients (43.8%). Brain, liver, and bone metastases were present in 18 (16.1%), 59 (52.7%), and 72 (64.3%) patients, respectively. Carboplatin was the predominant platinum agent, used in 94 patients (83.9%), whereas cisplatin was used in 18 patients (16.1%).

Baseline balance was assessed using standardized mean differences (SMDs). Most demographic and metastatic characteristics showed relatively small between-group differences, whereas the largest imbalances were observed for treatment year (SMD = 0.623), smoking cessation status (SMD = 0.500), LDH (SMD = 0.350), NSE (SMD = 0.275), platinum agent (SMD = 0.258), hypertension (SMD = 0.239), and PNI (SMD = 0.239).

### Overall survival

Kaplan-Meier analysis demonstrated longer OS in patients receiving first-line serplulimab plus chemotherapy than in those receiving chemotherapy alone (Figure 2A). Median OS was 18.69 months in the serplulimab plus chemotherapy group and 11.86 months in the chemotherapy-alone group. In the unadjusted Cox model, serplulimab plus chemotherapy was associated with a lower risk of death (HR = 0.49, 95% CI: 0.27-0.90; P = 0.021), with a log-rank P value of 0.017.

The estimated 12-month OS rates were 74.9% and 49.9% in the serplulimab plus chemotherapy and chemotherapy-alone groups, respectively; the corresponding 18-month OS rates were 70.5% and 35.0%. Estimates at later time points should be interpreted cautiously because of the limited number of patients remaining at risk.

After adjustment for age, ECOG performance status, liver metastasis, and brain metastasis, serplulimab plus chemotherapy remained associated with a lower risk of death (adjusted HR = 0.50, 95% CI: 0.27-0.93; P = 0.028) (Table 2). Exploratory extended models incorporating additional laboratory biomarkers are presented in Supplementary Table S3 and were not considered primary adjusted estimates.

### Real-world progression-free survival

Kaplan-Meier analysis showed a numerically longer rwPFS in patients receiving first-line serplulimab plus chemotherapy than in those receiving chemotherapy alone, although the difference was not statistically significant (Figure 2B). Median rwPFS was 10.64 months in the serplulimab plus chemotherapy group and 8.18 months in the chemotherapy-alone group. In the unadjusted Cox model, the HR for rwPFS was 0.76 (95% CI: 0.48-1.20; P = 0.244), with a log-rank P value of 0.243. After adjustment for age, ECOG performance status, liver metastasis, and brain metastasis, the association remained non-significant (adjusted HR = 0.78, 95% CI: 0.49-1.24; P = 0.290) (Table 2).

### Sensitivity analyses for overall survival

Multiple sensitivity analyses were performed to evaluate the consistency of the OS association (Figure 3). In the treatment-year adjusted model, serplulimab plus chemotherapy remained associated with a lower risk of death (HR = 0.51, 95% CI: 0.28-0.95; P = 0.033). When the prespecified clinical covariates and treatment year were included simultaneously, the effect estimate remained in the same direction but did not reach conventional statistical significance (HR = 0.55, 95% CI: 0.29-1.04; P = 0.065).

In the calendar-period restricted analysis including patients treated during 2022-2024, serplulimab plus chemotherapy was associated with longer OS (HR = 0.48, 95% CI: 0.26-0.89; P = 0.021). A similar association was observed after adjustment for age, ECOG performance status, liver metastasis, and brain metastasis within the restricted cohort (adjusted HR = 0.49, 95% CI: 0.26-0.91; P = 0.024).

Propensity score-based sensitivity analyses yielded directionally similar estimates. Overlap weighting produced an HR of 0.50 (95% CI: 0.28-0.89; P = 0.018), and stabilized trimmed IPTW produced an HR of 0.52 (95% CI: 0.29-0.93; P = 0.027).

Covariate balance before and after propensity score weighting is shown in Supplementary Figure S1. Before weighting, the largest imbalances among variables included in the reduced propensity score model were observed for ECOG performance status (SMD = 0.197), liver metastasis (SMD = 0.165), brain metastasis (SMD = 0.142), and age (SMD = 0.069). After overlap weighting, all included covariates were well balanced, with SMDs approaching zero. After stabilized trimmed IPTW, all included covariates had SMDs below 0.05. The effective sample size after overlap weighting was 78.44 in the chemotherapy-alone group and 29.76 in the serplulimab plus chemotherapy group.

### Exploratory clinical subgroup analyses for overall survival

Exploratory subgroup analyses for OS were performed according to age, sex, ECOG performance status, brain metastasis, liver metastasis, and bone metastasis (Figure 4). Treatment-effect estimates generally favored serplulimab plus chemotherapy across the examined subgroups. However, no statistically significant treatment-by-subgroup interaction was identified. The corresponding P-interaction values were 0.315 for age, 0.731 for sex, 0.514 for ECOG performance status, 0.772 for brain metastasis, 0.271 for liver metastasis, and 0.401 for bone metastasis. Given the limited sample size and number of events, these subgroup analyses should be considered exploratory, and non-significant interaction tests should not be interpreted as evidence of treatment-effect homogeneity.

Laboratory-marker subgroup analyses based on cohort-specific median cutoffs were reported only in the supplementary materials because dichotomization of continuous biomarkers may reduce information and reproducibility.

### Second-line and subsequent systemic therapy

Documented disease progression occurred in 54 of 82 patients in the chemotherapy-alone group and 22 of 30 patients in the serplulimab plus chemotherapy group. Among patients with documented progression, subsequent systemic therapy was identified from inpatient medication-order records in 42 of 54 (77.8%) patients in the chemotherapy-alone group and 9 of 22 (40.9%) patients in the serplulimab plus chemotherapy group. After supplementing medication-order data with available clinical documentation, the corresponding proportions were 43 of 54 (79.6%) and 9 of 22 (40.9%), respectively.

Among patients with documented progression, platinum-etoposide rechallenge was identified in 33 of 54 (61.1%) patients in the chemotherapy-alone group and 4 of 22 (18.2%) patients in the serplulimab plus chemotherapy group. Irinotecan-containing therapy was documented in 12 of 54 (22.2%) and 3 of 22 (13.6%) patients, respectively, while subsequent immune checkpoint inhibitor therapy was documented in 15 of 54 (27.8%) and 8 of 22 (36.4%) patients. Three or more lines of systemic therapy were documented in 10 of 54 (18.5%) and 2 of 22 (9.1%) patients, respectively.

No subsequent systemic therapy was identified in inpatient medication-order records for 12 of 54 (22.2%) patients in the chemotherapy-alone group and 13 of 22 (59.1%) patients in the serplulimab plus chemotherapy group. Detailed post-progression treatment patterns are summarized in Supplementary Table S1. Because outpatient oral anticancer therapies were incompletely captured, agent-specific treatment exposure derived from inpatient medication-order records may underestimate actual post-progression treatment.

### Exploratory safety assessment

Because prospectively collected CTCAE-graded adverse events and standardized irAE assessments were unavailable, safety findings were summarized descriptively using predefined record-based proxies. A systemic corticosteroid-based irAE proxy was identified in 2 of 82 patients (2.4%) in the chemotherapy-alone group and 2 of 30 patients (6.7%) in the serplulimab plus chemotherapy group. Explicitly documented strict safety events were identified in 79 of 82 patients (96.3%) and 6 of 30 patients (20.0%), respectively. This marked between-group difference was considered indicative of differential documentation completeness rather than a valid comparison of toxicity incidence. Among the six serplulimab-treated patients with explicitly documented strict safety events, five had pulmonary events described in the source records as “pulmonary fibrosis” and one had documented immune-mediated myocardial injury. The pulmonary events were not reclassified as immune-related pneumonitis because standardized causal adjudication was unavailable. Treatment interruption, permanent treatment discontinuation, toxicity-related hospitalization, and fatal toxicity could not be reliably ascertained from the available records. Detailed exploratory safety findings are summarized in Supplementary Table S2.

## Discussion

In this single-center real-world cohort of patients with ES-SCLC, first-line serplulimab plus chemotherapy was associated with longer OS than chemotherapy alone, whereas the difference in rwPFS was numerically favorable but did not reach statistical significance. The association with longer OS was directionally consistent across the primary multivariable model and several complementary sensitivity analyses, including treatment-year adjustment, restriction to patients treated during 2022-2024, overlap weighting, and stabilized trimmed IPTW. However, the combined clinical plus treatment-year adjusted model yielded an HR of 0.55 (95% CI, 0.29-1.04; P = 0.065), indicating that the magnitude and statistical precision of the observed association were sensitive to calendar-time adjustment. These findings therefore support a real-world association between first-line serplulimab-based chemoimmunotherapy and longer survival but should not be interpreted as establishing a definitive causal treatment effect.

The observed OS difference is clinically relevant in the context of ES-SCLC, a disease characterized by rapid progression, early metastatic dissemination, and poor long-term survival. Platinum-etoposide chemotherapy has historically been the standard first-line treatment, although durable disease control and long-term survival with chemotherapy alone remain limited. The incorporation of immune checkpoint inhibitors has substantially changed the first-line treatment landscape. Serplulimab, a programmed cell death protein 1 (PD-1) inhibitor, demonstrated a significant survival benefit when combined with chemotherapy in treatment-naïve ES-SCLC in the phase 3 ASTRUM-005 trial and subsequent analyses 13-15. Our findings extend these trial observations by providing comparative evidence from routine clinical practice, where patient characteristics, treatment decisions, supportive care, and follow-up patterns may differ from those in randomized clinical trials 20-26.

A notable finding was the discrepancy between OS and rwPFS. Although a statistically significant association with longer OS was observed, rwPFS showed only a numerical improvement. Several factors may contribute to this pattern. First, rwPFS in retrospective studies is inherently more susceptible to measurement variability than protocol-defined PFS because imaging intervals, assessment timing, and documentation of progression are not standardized 27,28. In the present study, progression was abstracted from radiology reports and treating-physician documentation, and RECIST version 1.1 criteria were applied only when sufficient measurable-lesion information was explicitly available 29. Variation in imaging intervals and surveillance intensity may therefore have influenced the timing of documented progression. Second, survival effects associated with immune checkpoint inhibition may not always be fully reflected by early progression-based endpoints 40,41. Third, post-progression treatment and supportive care may influence OS independently of the timing of first documented progression. Accordingly, the rwPFS findings should be interpreted cautiously, and greater weight should be placed on OS as the primary endpoint of this study.

Post-progression treatment patterns also differed between the treatment groups. Among patients with documented progression, subsequent systemic therapy was identified from inpatient medication-order records in 77.8% of patients in the chemotherapy-alone group and 40.9% of those receiving serplulimab plus chemotherapy; after supplementation with available clinical documentation, the corresponding proportions were 79.6% and 40.9%. Subsequent immune checkpoint inhibitor therapy was documented in 27.8% and 36.4% of patients, respectively. These findings suggest that the observed OS difference cannot be simply attributed to greater access to subsequent systemic therapy in the serplulimab group. Nevertheless, subsequent treatment is a post-baseline variable influenced by progression, post-progression survival, performance status, treatment response, and subsequent clinical decision-making. These descriptive comparisons therefore cannot determine the causal contribution of post-progression therapy to OS. In addition, outpatient oral anticancer therapies were incompletely captured, and specific post-progression treatments may consequently have been under-ascertained.

Potential calendar-time confounding is another important consideration. Patients in the chemotherapy-alone group were treated across a broader historical period, whereas patients receiving serplulimab plus chemotherapy were concentrated in the more contemporary treatment era. During this interval, changes in supportive care, access to subsequent systemic therapies, the integration and timing of thoracic radiotherapy, radiologic staging, and imaging surveillance may have influenced outcomes independently of first-line treatment. Consistent with this concern, simultaneous adjustment for the prespecified clinical covariates and treatment year attenuated the treatment estimate to HR = 0.55 (95% CI, 0.29-1.04; P = 0.065), with the confidence interval crossing unity. Analyses restricted to patients treated during 2022-2024 yielded directionally similar estimates and remained statistically significant both before and after parsimonious clinical adjustment. However, neither statistical adjustment nor calendar-period restriction can fully eliminate residual confounding from unmeasured temporal changes in clinical management. The observed OS association should therefore be interpreted in the context of this remaining uncertainty.

The analytical strategy was deliberately parsimonious to reduce the risk of overfitting in a modest-sized retrospective cohort. The primary multivariable Cox model was restricted to prespecified clinical covariates rather than incorporating numerous laboratory biomarkers. Similarly, the reduced propensity score model included age, ECOG performance status, liver metastasis, and brain metastasis to limit model complexity and unstable weighting. Overlap weighting was selected as the principal propensity score-based sensitivity approach because it down-weights patients with extreme treatment probabilities and focuses estimation on patients with greater clinical equipoise 35-37. Covariate balance improved substantially after weighting, while effective sample size was largely preserved. Nevertheless, propensity score methods can address only measured covariates and should be regarded as complementary sensitivity analyses rather than definitive causal estimates 33,34,38.

Exploratory subgroup analyses generally showed treatment-effect estimates in favor of serplulimab plus chemotherapy across the selected clinical subgroups. However, no statistically significant treatment-by-subgroup interaction was identified. Given the limited sample size and number of events, these analyses had limited power to detect genuine effect modification. Accordingly, non-significant interaction tests should not be interpreted as evidence of treatment-effect homogeneity, and apparent variation in subgroup-specific hazard ratios may reflect sparse events and sampling variability. Laboratory-marker subgroup analyses based on cohort-specific median cutoffs were therefore retained only as supplementary exploratory analyses because dichotomization of continuous biomarkers may reduce information and reproducibility.

This study has several strengths. It addresses a clinically relevant question regarding the comparative effectiveness of first-line serplulimab-based chemoimmunotherapy in routine clinical practice and directly compares outcomes with a chemotherapy-alone comparison cohort treated at the same institution. In addition, multiple complementary analytical approaches, including parsimonious multivariable adjustment, treatment-year adjustment, calendar-period restriction, overlap weighting, stabilized trimmed IPTW, and interaction testing—were used to examine the consistency and uncertainty of the observed OS association.

Several limitations should also be acknowledged. First, this was a single-center retrospective study with a limited sample size, particularly in the serplulimab plus chemotherapy group, which included only 30 patients and 13 deaths. The limited number of events constrained the precision and statistical power of the multivariable Cox models, propensity score analyses, and treatment-by-subgroup interaction tests. Although the primary Cox and propensity score models were deliberately kept parsimonious to reduce overfitting, residual model instability cannot be excluded. Second, residual confounding remains possible because treatment allocation was not randomized, and calendar-time effects are of particular concern given the different treatment periods of the two groups. The attenuation of the treatment estimate in the clinical plus treatment-year adjusted model further underscores this uncertainty. Third, rwPFS was determined from routine clinical documentation and imaging reports rather than protocol-scheduled tumor assessments or blinded independent central review. Variation in imaging intervals and surveillance intensity may therefore have affected the timing of documented progression. Fourth, prospectively collected and systematically CTCAE-graded adverse-event data were unavailable. Although predefined record-based safety proxies, including a corticosteroid-based proxy for potentially clinically significant immune-mediated toxicity, were evaluated, adverse-event documentation was incomplete and uneven across treatment groups. Treatment interruption, permanent discontinuation, toxicity-related hospitalization, and fatal toxicity could not be reliably ascertained. Therefore, the safety findings should be interpreted as descriptive clinical signals rather than as a complete estimate of treatment-related toxicity or irAE incidence. Finally, the single-center design may limit external generalizability, and the findings require validation in larger multicenter prospective cohorts with standardized imaging assessment, adverse-event collection, and follow-up procedures.

## Conclusions

In this single-center real-world cohort of patients with ES-SCLC, first-line serplulimab plus chemotherapy was associated with longer OS than chemotherapy alone, whereas the difference in rwPFS was not statistically significant. The OS association was directionally consistent across multiple complementary sensitivity analyses, although its magnitude and precision were sensitive to calendar-time adjustment. Given the limited sample size, potential residual and calendar-time confounding, and incomplete standardized safety assessment, these findings should be interpreted cautiously and regarded as hypothesis-generating. Validation in larger prospective multicenter cohorts is warranted.

## Supplementary Material

Supplementary tables.

## Figures and Tables

**Figure 1:**
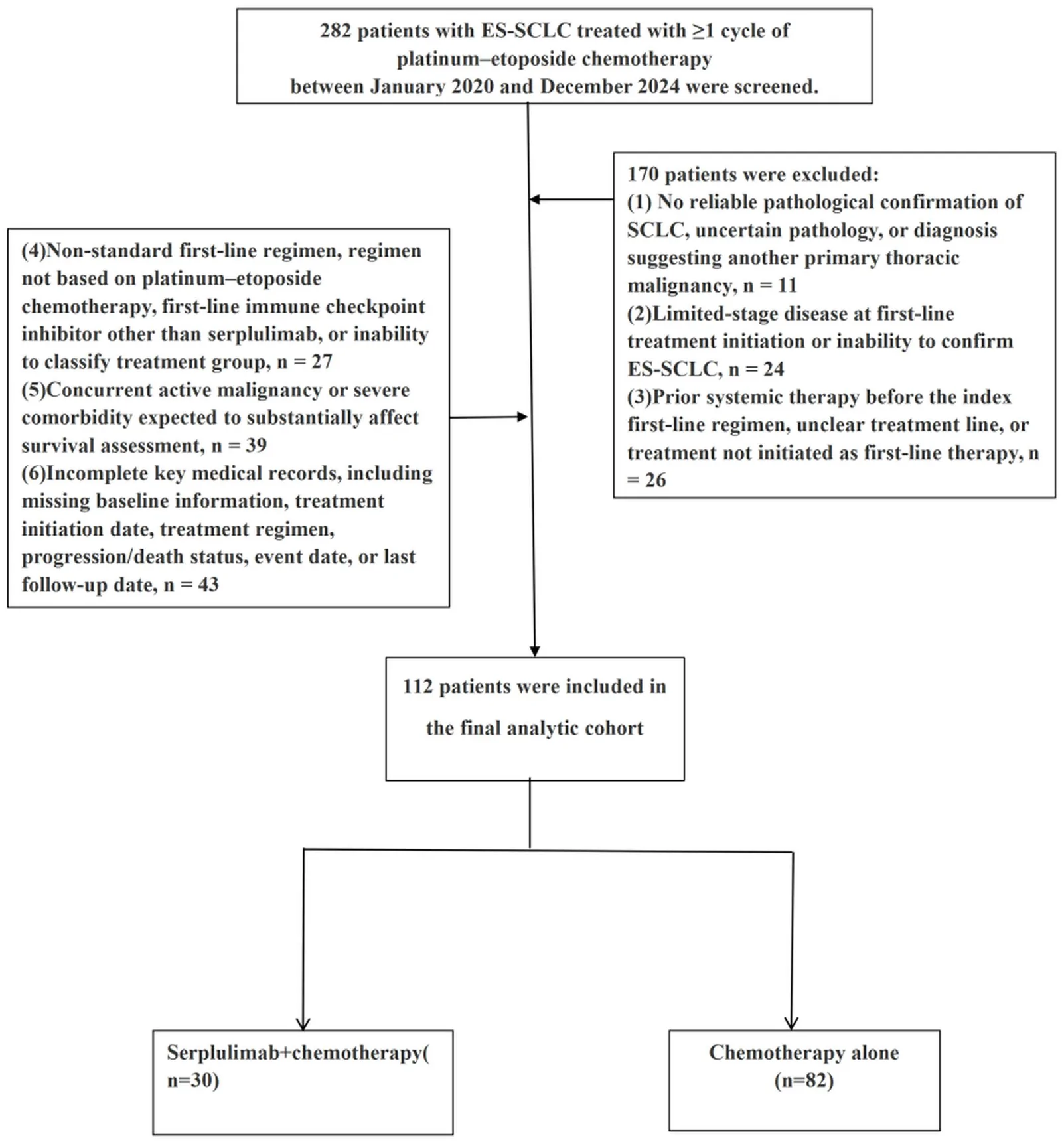
** Flow chart of patient selection.** A total of 282 patients with extensive-stage small-cell lung cancer (ES-SCLC) who received at least one cycle of platinum-etoposide chemotherapy between January 2020 and December 2024 were screened. After exclusion of 170 patients who did not meet the eligibility criteria or had incomplete key records, 112 patients were included in the final analytic cohort, including 30 treated with serplulimab plus chemotherapy and 82 treated with chemotherapy alone. ES-SCLC, extensive-stage small-cell lung cancer.

**Figure 2:**
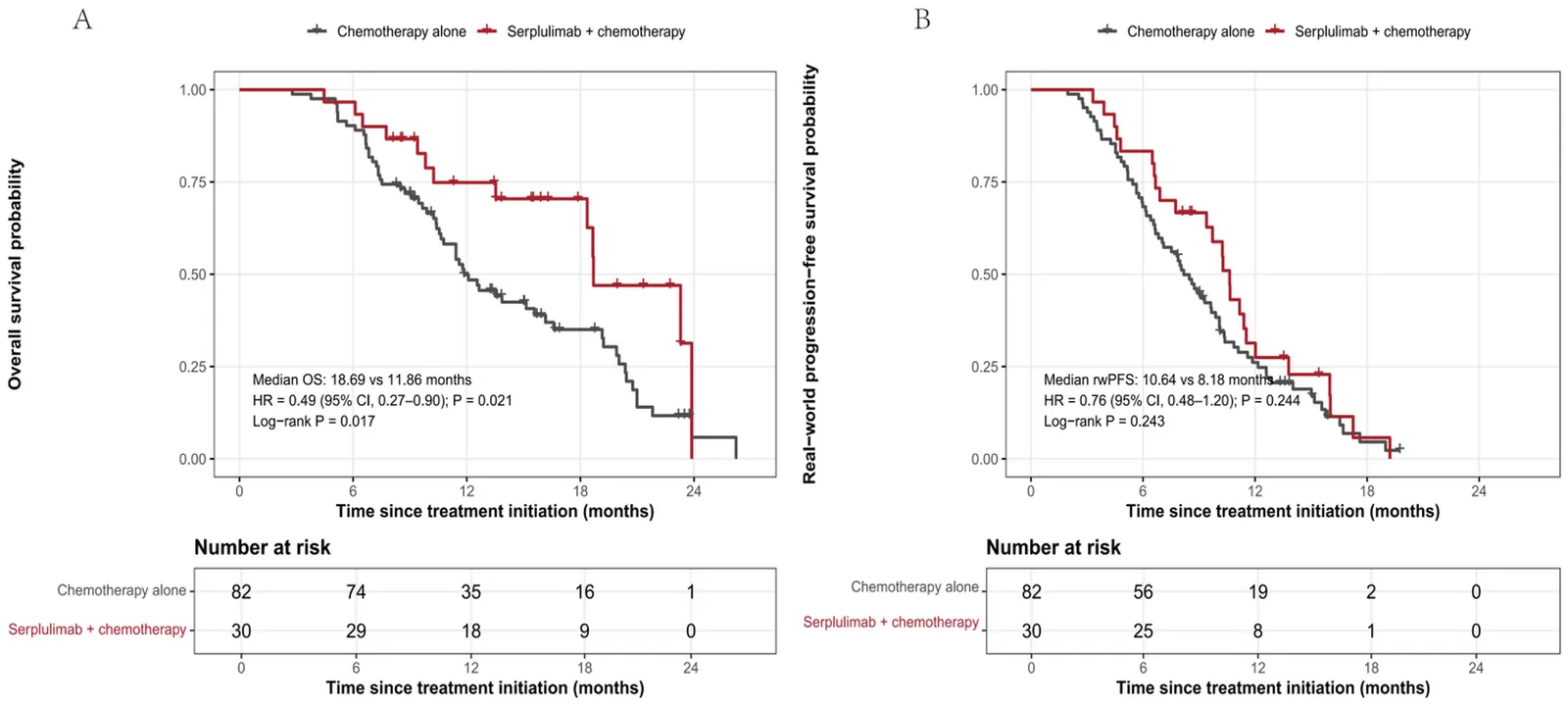
** Kaplan-Meier curves for overall survival and real-world progression-free survival according to first-line treatment group.** (A) Overall survival in patients receiving serplulimab plus chemotherapy versus chemotherapy alone. (B) Real-world progression-free survival in patients receiving serplulimab plus chemotherapy versus chemotherapy alone. Hazard ratios and 95% confidence intervals were estimated using unadjusted Cox proportional hazards models. Log-rank P values are shown in the plots. OS, overall survival; rwPFS, real-world progression-free survival; HR, hazard ratio; CI, confidence interval.

**Figure 3:**
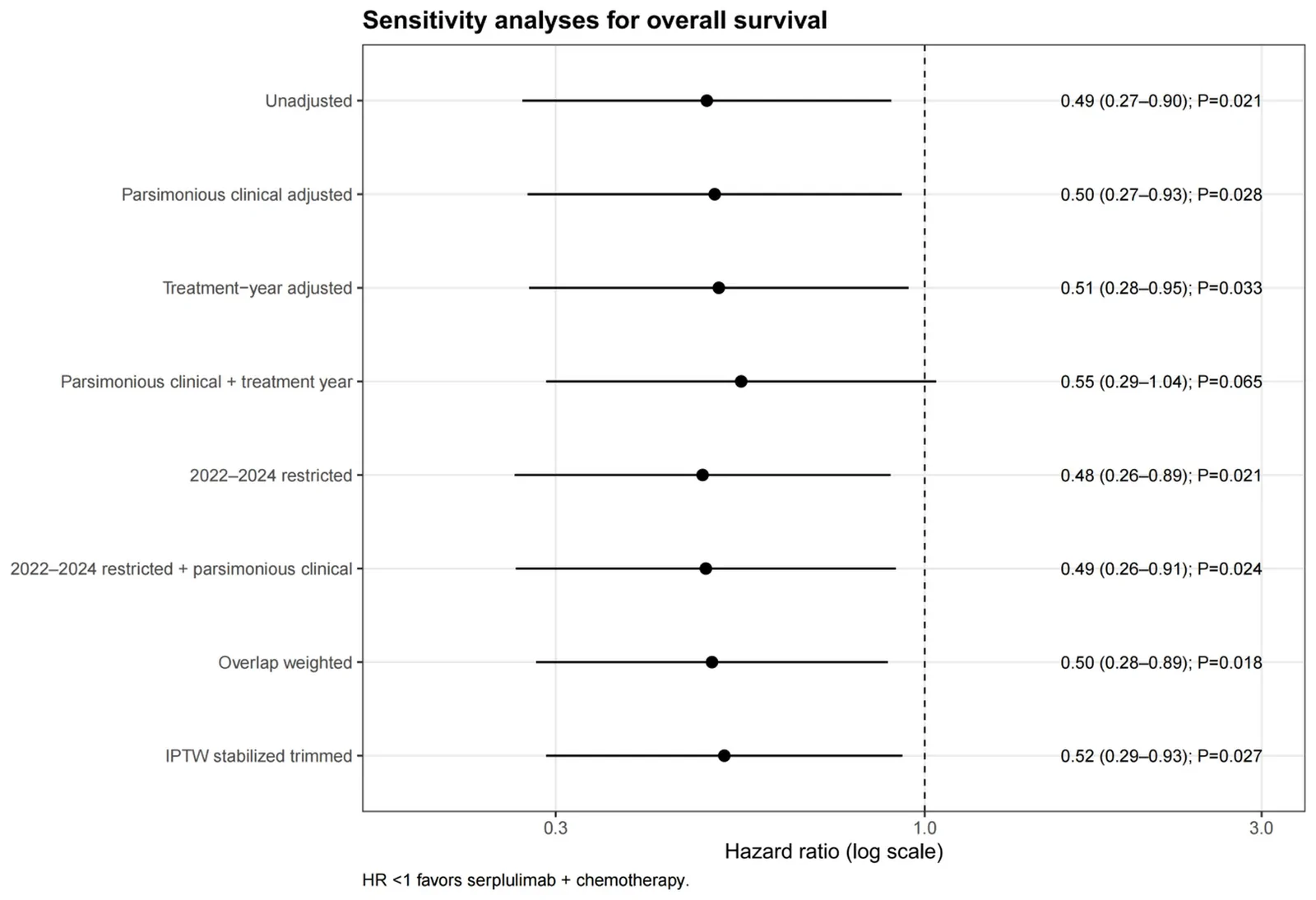
** Sensitivity analyses for overall survival.** Forest plot showing hazard ratios and 95% confidence intervals for OS across the primary analysis and prespecified sensitivity analyses. The primary adjusted model was a parsimonious clinical model including age, ECOG performance status, liver metastasis, and brain metastasis. Propensity score-based analyses used a reduced clinical propensity score model and were considered sensitivity analyses. Hazard ratios <1 favor serplulimab plus chemotherapy. OS, overall survival; HR, hazard ratio; CI, confidence interval; ECOG, Eastern Cooperative Oncology Group; IPTW, inverse probability of treatment weighting.

**Figure 4:**
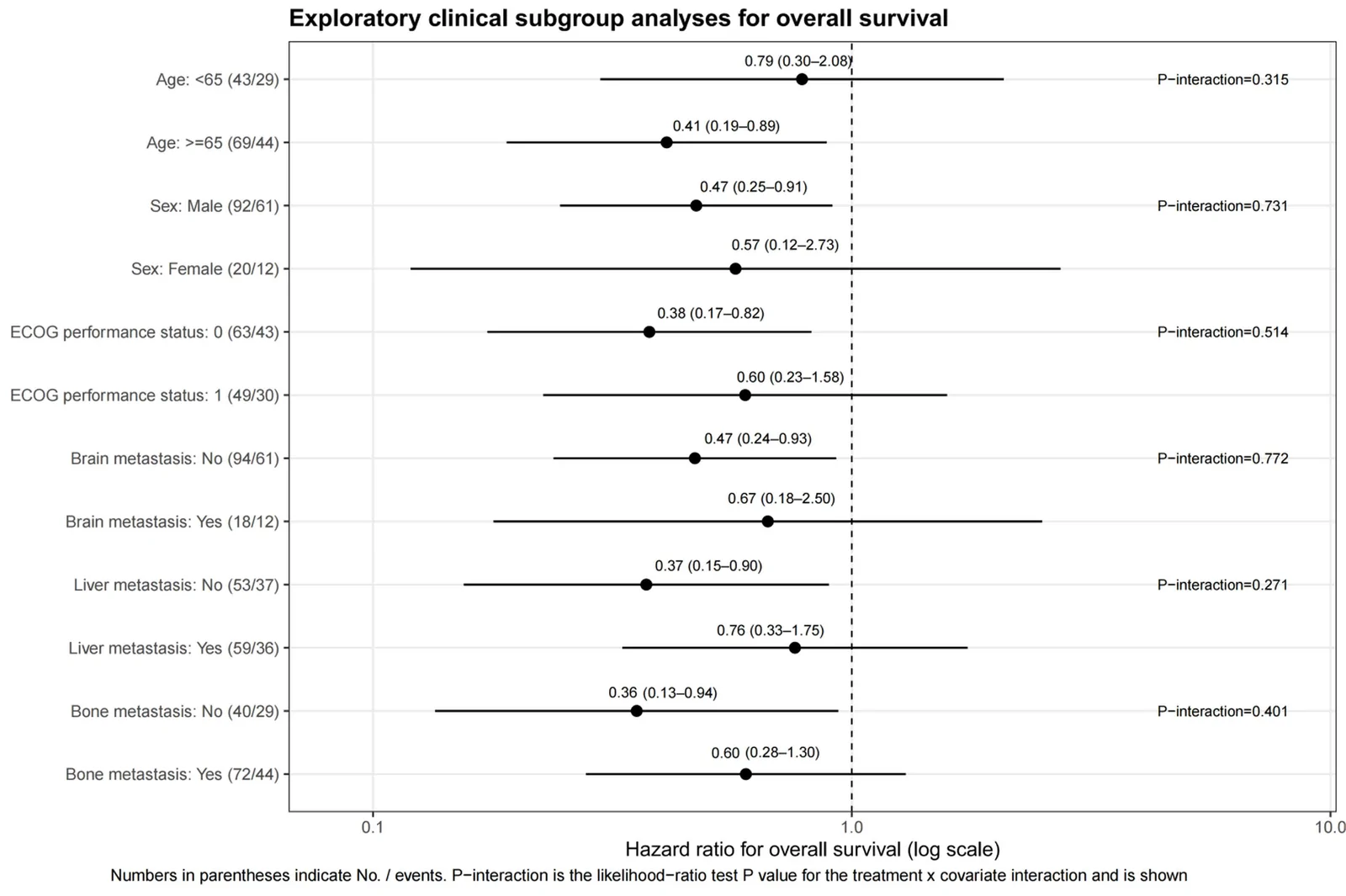
** Exploratory clinical subgroup analyses for overall survival.** Forest plot showing hazard ratios and 95% confidence intervals for OS across selected clinical subgroups. Treatment-by-subgroup interaction was assessed using likelihood-ratio tests, with one P-interaction value reported for each covariate. Laboratory-marker subgroup analyses based on cohort-specific median cutoffs were excluded from the main figure and reported separately as supplementary exploratory analyses. Given the limited sample size and number of events in some strata, subgroup findings should be interpreted cautiously. OS, overall survival; HR, hazard ratio; CI, confidence interval; ECOG, Eastern Cooperative Oncology Group.

**Table 1:** Baseline characteristics of patients according to first-line treatment group.

Characteristic	Overall (N = 112)	Chemotherapy alone (n = 82)	Serplulimab + chemotherapy (n = 30)	SMD
Age, years	68 [62, 74]	68 [61, 74]	68 [63, 72]	0.069
Male sex, n (%)	92 (82.1)	66 (80.5)	26 (86.7)	0.167
ECOG performance status, n (%)				0.197
0	63 (56.2)	44 (53.7)	19 (63.3)	
1	49 (43.8)	38 (46.3)	11 (36.7)	
Brain metastasis, n (%)	18 (16.1)	12 (14.6)	6 (20.0)	0.142
Liver metastasis, n (%)	59 (52.7)	45 (54.9)	14 (46.7)	0.165
Bone metastasis, n (%)	72 (64.3)	53 (64.6)	19 (63.3)	0.027
Pack-years	30.0 [15.0, 50.0]	30.0 [15.0, 50.0]	30.0 [22.5, 50.0]	0.061
Quit smoking, n (%)	27 (24.1)	24 (29.3)	3 (10.0)	0.500
Hypertension, n (%)	50 (44.6)	34 (41.5)	16 (53.3)	0.239
Coronary disease, n (%)	20 (17.9)	14 (17.1)	6 (20.0)	0.075
Diabetes, n (%)	18 (16.1)	13 (15.9)	5 (16.7)	0.022
Platinum agent, n (%)				0.258
Carboplatin	94 (83.9)	71 (86.6)	23 (76.7)	
Cisplatin	18 (16.1)	11 (13.4)	7 (23.3)	
Treatment year, n (%)				0.623
2020	1 (0.9)	1 (1.2)	0 (0.0)	
2021	8 (7.1)	8 (9.8)	0 (0.0)	
2022	26 (23.2)	22 (26.8)	4 (13.3)	
2023	39 (34.8)	27 (32.9)	12 (40.0)	
2024	38 (33.9)	24 (29.3)	14 (46.7)	
LDH, U/L	233.5 [191.0, 292.5]	239.5 [199.0, 296.0]	220.5 [188.0, 286.0]	0.350
NSE, ng/mL	40.2 [22.1, 84.5]	42.9 [24.1, 84.8]	34.2 [19.6, 65.5]	0.275
Albumin, g/L	40.1 [37.3, 42.5]	40.3 [36.6, 42.6]	39.5 [37.7, 42.2]	0.085
NLR	2.63 [2.09, 3.78]	2.68 [2.09, 3.93]	2.51 [2.04, 3.50]	0.033
PLR	153.1 [114.3, 210.9]	157.2 [118.3, 213.8]	141.3 [105.5, 206.3]	0.160
SII	655.0 [438.3, 1009.5]	669.7 [442.0, 1126.7]	608.8 [424.1, 833.2]	0.038
PNI	48.3 [44.2, 52.1]	48.3 [43.9, 51.6]	48.0 [45.5, 53.8]	0.239
ProGRP, pg/mL	843.7 [152.0, 2768.0]	895.8 [188.8, 2868.0]	706.5 [83.2, 2476.0]	0.107

Data are presented as median [interquartile range] for continuous variables and n (%) for categorical variables. Baseline balance between treatment groups was assessed using standardized mean differences (SMDs). P values were not reported for baseline comparisons because SMDs provide a sample-size-independent measure of covariate imbalance. An absolute SMD >0.10 was considered indicative of meaningful imbalance. ECOG, Eastern Cooperative Oncology Group; LDH, lactate dehydrogenase; NSE, neuron-specific enolase; NLR, neutrophil-to-lymphocyte ratio; PLR, platelet-to-lymphocyte ratio; SII, systemic immune-inflammation index; PNI, prognostic nutritional index; ProGRP, pro-gastrin-releasing peptide. NLR, PLR, SII, and PNI are derived indices and are presented without physical units.

**Table 2:** Treatment effect of serplulimab plus chemotherapy versus chemotherapy alone on overall survival and real-world progression-free survival.

Endpoint	Model	HR (95% CI)	P value
OS	Unadjusted	0.49 (0.27-0.90)	0.021
OS	Parsimonious clinical adjusted	0.50 (0.27-0.93)	0.028
rwPFS	Unadjusted	0.76 (0.48-1.20)	0.244
rwPFS	Parsimonious clinical adjusted	0.78 (0.49-1.24)	0.290

Hazard ratios were estimated using Cox proportional hazards models, with chemotherapy alone as the reference group. The parsimonious adjusted model included treatment group, age, ECOG performance status, liver metastasis, and brain metastasis. Model complexity was deliberately restricted to reduce the risk of overfitting given the limited sample size and number of events. Hazard ratios < 1 favor serplulimab plus chemotherapy. OS, overall survival; rwPFS, real-world progression-free survival; HR, hazard ratio; CI, confidence interval; ECOG, Eastern Cooperative Oncology Group.

## Abbreviations

ES-SCLC: extensive-stage small-cell lung cancer
SCLC: small-cell lung cancer
OS: overall survival
rwPFS: real-world progression-free survival
PFS: progression-free survival
ECOG: Eastern Cooperative Oncology Group
VALSG: Veterans Administration Lung Study Group
EMR: electronic medical record
RECIST: Response Evaluation Criteria in Solid Tumors
CTCAE: Common Terminology Criteria for Adverse Events
irAE: immune-related adverse event
SMD: standardized mean difference
HR: hazard ratio
CI: confidence interval
LDH: lactate dehydrogenase
NSE: neuron-specific enolase
NLR: neutrophil-to-lymphocyte ratio
PLR: platelet-to-lymphocyte ratio
SII: systemic immune-inflammation index
PNI: prognostic nutritional index
ProGRP: pro-gastrin-releasing peptide
IPTW: inverse probability of treatment weighting
PD-1: programmed cell death protein 1
PD-L1: programmed death-ligand 1
STROBE: Strengthening the Reporting of Observational Studies in Epidemiology
