# Supplementary material for: Real-World Comparative Survival Outcomes of First-Line Serplulimab Plus Chemotherapy in Extensive-Stage Small-Cell Lung Cancer: A Single-Center Retrospective Cohort Study
Source: J Cancer. 2026 Oct 16;17(11):1915–26. doi: 10.7150/jca.140183 (PMC13686793; doi:10.7150/jca.140183)
Supplement: Supplementary file 1 [file jcav17p1915s1.pdf]

Supplementary Materials

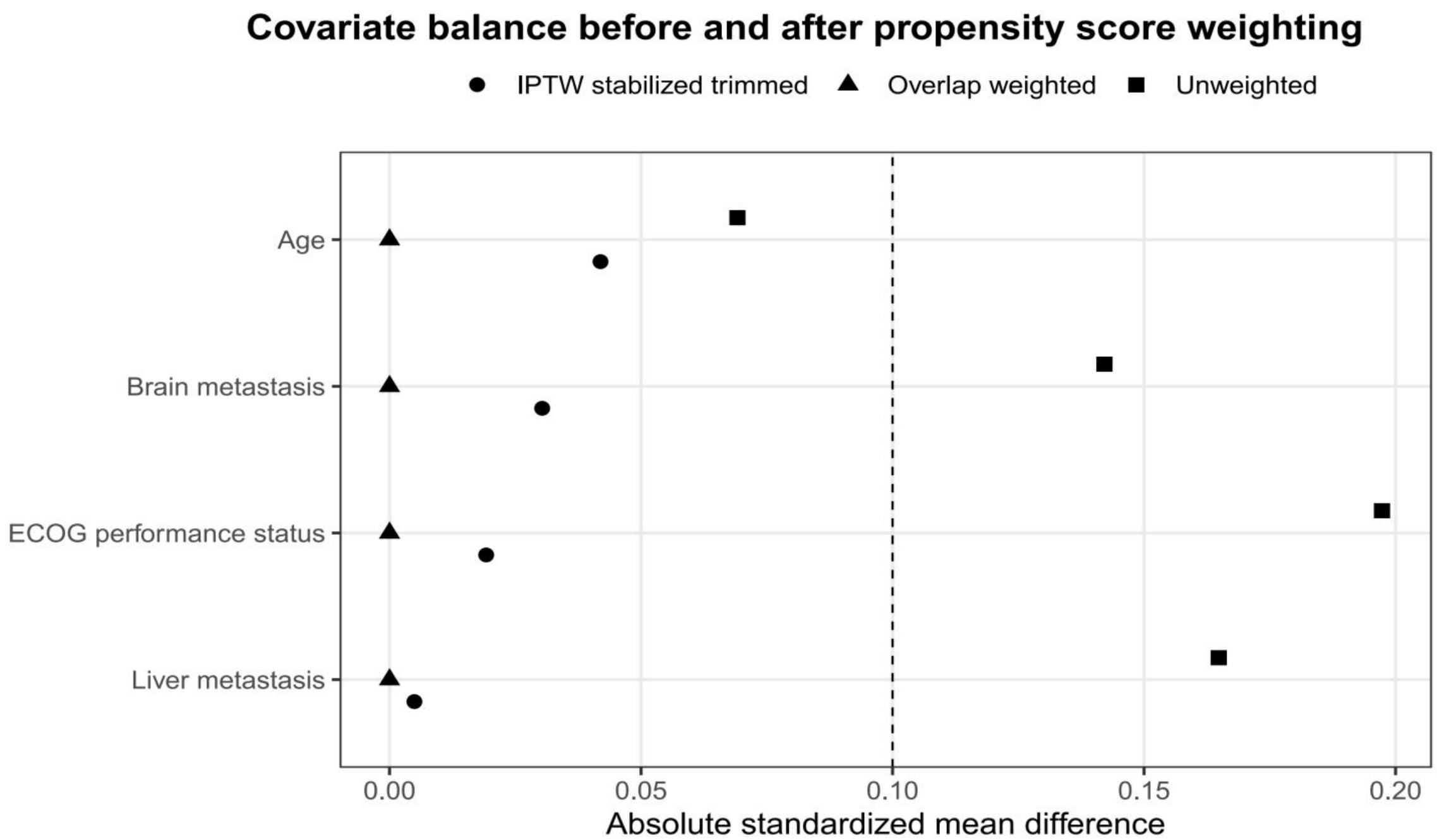

Supplementary Figure S1. Love plot showing absolute standardized mean differences (SMDs) for covariates included in the reduced propensity score model before weighting, after overlap weighting, and after stabilized trimmed inverse probability of treatment weighting (IPTW). The dashed horizontal line indicates SMD = 0.1.

Supplementary Table S1. Second-line and subsequent systemic therapy among patients with documented disease progression.

Subsequent treatment	Chemotherapy alone (n = 54)	Serplulimab + chemotherapy (n = 22)
Patients with documented progression	54	22
Evaluable for subsequent treatment	54	22
Any subsequent systemic therapy, inpatient medication orders	42 (77.8%)	9 (40.9%)
Any subsequent systemic therapy, orders + clinical documentation	43 (79.6%)	9 (40.9%)
Platinum–etoposide rechallenge	33 (61.1%)	4 (18.2%)
Etoposide rechallenge	27 (50.0%)	3 (13.6%)
Irinotecan-containing therapy	12 (22.2%)	3 (13.6%)
Subsequent immune checkpoint inhibitor therapy	15 (27.8%)	8 (36.4%)
≥3 systemic treatment lines	10 (18.5%)	2 (9.1%)
No subsequent systemic therapy identified in inpatient medication-order records	12 (22.2%)	13 (59.1%)
No subsequent systemic therapy identified by combined ascertainment	11 (20.4%)	13 (59.1%)
Topotecan documented in inpatient medication orders	0 (0.0%)	0 (0.0%)
Lurbinectedin documented in inpatient medication orders	0 (0.0%)	0 (0.0%)
Anlotinib documented in inpatient medication orders	0 (0.0%)	0 (0.0%)

Note: Data are presented as n (%), except for the first two rows. Denominators are patients with documented disease progression. Treatment categories were not mutually exclusive. Maintenance serplulimab administered before progression as part of first-line treatment was not classified as subsequent-line therapy. Agent-specific treatment exposure was primarily derived from inpatient medication-order records. Because outpatient oral anticancer therapies were incompletely captured, absence of a medication order should not be interpreted as definitive absence of treatment.

Supplementary Table S2. Exploratory record-based safety assessment according to first-line treatment group.

Record-based safety item	Chemotherapy alone (n = 82)	Serplulimab + chemotherapy (n = 30)
Any explicitly documented strict safety event	79 (96.3%)	6 (20.0%)
Pulmonary event described as "pulmonary fibrosis"	0 (0.0%)	5 (16.7%)
Immune-mediated myocardial injury, explicitly documented	0 (0.0%)	1 (3.3%)
Thyroid event	7 (8.5%)	0 (0.0%)
Systemic corticosteroid-based irAE proxy	2 (2.4%)	2 (6.7%)
Treatment interruption because of toxicity	Not reliably captured	Not reliably captured
Permanent treatment discontinuation because of toxicity	Not reliably captured	Not reliably captured
Toxicity-related hospitalization	Not reliably captured	Not reliably captured
Fatal toxicity	Not reliably captured	Not reliably captured

Note: This exploratory analysis was based on retrospective clinical documentation and was not intended to estimate the incidence of treatment-related adverse events or immune-related adverse events (irAEs). Prospectively collected CTCAE-graded adverse-event data and standardized irAE adjudication were unavailable. The marked difference in the frequency of explicitly documented safety events between treatment groups likely reflects differential documentation completeness and should not be interpreted as a valid comparison of toxicity incidence. Pulmonary events were retained using the source-record term "pulmonary fibrosis" and were not reclassified as immune-related pneumonitis because standardized causal adjudication was unavailable. Treatment interruption, permanent treatment discontinuation, toxicity-related hospitalization, and fatal toxicity could not be reliably ascertained and were not coded as absent when undocumented.

Supplementary Table S3. Exploratory extended Cox regression analyses for overall survival and real-world progression-free survival.

Endpoint	Model	HR	95% CI	P value
OS	Exploratory extended clinical + laboratory adjusted model	0.52	0.28–0.97	0.039
rwPFS	Exploratory extended clinical + laboratory adjusted model	0.76	0.47–1.22	0.249

Note: Hazard ratios compare serplulimab plus chemotherapy with chemotherapy alone as the reference group. Extended models were exploratory and were not considered the primary adjusted analyses. The extended model incorporated the prespecified clinical covariates together with selected laboratory biomarkers, including LDH, NSE, SII, and PNI. OS, overall survival; rwPFS, real-world progression-free survival; HR, hazard ratio; CI, confidence interval; LDH, lactate dehydrogenase; NSE, neuron-specific enolase; SII, systemic immune-inflammation index; PNI, prognostic nutritional index.

Supplementary Table S4. Exploratory laboratory-marker subgroup analyses for overall survival.

Marker / subgroup	Level	n	Events	HR (95% CI)	Within-stratum P value	P-interaction
LDH, cutoff 233.5 U/L	Low	56	33	0.31 (0.12–0.80)	0.016	0.137
	High	56	40	0.75 (0.34–1.63)	0.464	
NSE, cutoff 40.2 ng/mL	Low	56	34	0.56 (0.24–1.28)	0.169	0.798
	High	56	39	0.46 (0.19–1.11)	0.085	
SII, cutoff 655	Low	56	35	0.43 (0.19–1.01)	0.052	0.812
	High	56	38	0.50 (0.21–1.20)	0.121	
PNI, cutoff 48.275	High	56	36	0.68 (0.30–1.57)	0.366	0.256
	Low	56	37	0.36 (0.15–0.87)	0.023	

Note: Cutoffs were cohort-specific medians. Hazard ratios compare serplulimab plus chemotherapy with chemotherapy alone within each stratum. Within-stratum P values were derived from subgroup-specific Cox models. P-interaction values were derived from likelihood-ratio tests comparing Cox models with and without the treatment-by-subgroup interaction term and apply to both levels of each marker. These analyses were exploratory and underpowered; statistically significant within-stratum estimates should not be interpreted as evidence of predictive treatment effects in the absence of a significant interaction. LDH, lactate dehydrogenase; NSE, neuron-specific enolase; SII, systemic immune-inflammation index; PNI, prognostic nutritional index; HR, hazard ratio; CI, confidence interval.
